# Raman Spectrum of the Li_2_SO_4_-MgSO_4_-H_2_O System: Excess Spectrum and Hydration Shell Spectrum

**DOI:** 10.3390/molecules28217356

**Published:** 2023-10-31

**Authors:** Haiwen Ge, Min Wang

**Affiliations:** 1 Key Laboratory of Comprehensive and Highly Efficient Utilization of Salt Lake Resources, Qinghai Institute of Salt Lakes, Chinese Academy of Sciences, Xining 810008, China; 2 Qinghai Provincial Key Laboratory of Resources and Chemistry of Salt Lakes, Xining 810008, China

**Keywords:** excess Raman spectroscopy, ratio spectra, hydration shell, hydration number, lithium–magnesium separation system

## Abstract

Lithium, as a green energy metal used to promote world development, is an important raw material for lithium-ion, lithium–air, and lithium–sulfur batteries. It is challenging to directly extract lithium resources from brine with a high Mg/Li mass ratio. The microstructure study of salt solutions provides an important theoretical basis for the separation of lithium and magnesium. The changes in the hydrogen bond network structure and ion association of the Li_2_SO_4_ aqueous solution and Li_2_SO_4_-MgSO_4_-H_2_O mixed aqueous solution were studied by Raman spectroscopy. The SO_4_^2−^ fully symmetric stretching vibration peak at 940~1020 cm^−1^ and the O-H stretching vibration peak at 2800~3800 cm^−1^ of the Li_2_SO_4_ aqueous solution at room temperature were studied by Raman spectroscopy and excess spectroscopy. According to the peak of the O-H stretching vibration spectrum, with an increase in the mass fraction of the Li_2_SO_4_ solution, the proportion of DAA-type and DDAA-type hydrogen bonds at low wavenumbers decreases gradually, while the proportion of DA-type hydrogen bonds at 3300 cm^−1^ increases. When the mass fraction is greater than 6.00%, this proportion increases sharply. Although the spectra of hydrated water molecules and bulk water molecules are different, the spectra of the two water molecules seriously overlap. The spectrum of the anion hydration shell in a solution can be extracted via spectrum division. By analyzing the spectra of these hydration shells, the interaction between the solute and water molecules, the structure of the hydration shell and the number of water molecules are obtained. For the same ionic strength solution, different cationic salts have different hydration numbers of anions, indicating that there is a strong interaction between ions in a strong electrolytic solution, which will lead to ion aggregation and the formation of ion pairs. When the concentration of salt solution increases, the hydration number decreases rapidly, indicating that the degree of ion aggregation increases with increasing concentration.

## 1. Introduction

Lithium, as a green energy metal that promotes the development of the world, is widely used in batteries, nuclear reactors, aerospace alloys, ceramics, medicine, metallurgy and additives due to its unique physical and chemical properties [1,2]. Lithium is also an important raw material for lithium-ion and next-generation batteries (such as lithium–air and lithium–sulfur) [3,4,5]. According to USGS 2018 [6], these batteries will consume 40% of the total lithium production. The 2019 Chinese government work report clearly pointed out that “facilitating the building of charging and hydrogenation facilities” would greatly promote the market share of new energy industries such as rechargeable lithium-ion and hydrogen fuel cells. With the rapid development of rechargeable new energy vehicles, the demand for lithium-ion batteries is increasing, and a shortage of lithium supply is inevitable [7,8]. Therefore, increasing attention has been given to the exploitation and extraction of lithium resources. The lithium storage in China is approximately 7 million tons, of which more than 80% is salt lake brine (mainly salt lake brine with a high Mg/Li ratio) [9]. With the increasing demand for lithium, lithium extraction from salt lake brine has become an inevitable trend. In the periodic table of elements, Mg and Li are diagonal, and the chemical properties of Mg and Li are similar. Therefore, the direct extraction of lithium from high-Mg/Li-ratio brine is challenging, and a large amount of magnesium resources will be wasted in the extraction process [10,11]. At present, the main methods for lithium extraction from brine include precipitation, solvent extraction, adsorption and membrane methods [12,13,14].

The separation method for lithium and magnesium involves the transformation process of the ionic hydration structure (ion dehydration and rehydration) in the solution. The microscopic hydration structure of an Li-Mg mixed salt solution will provide an important theoretical basis for Li-Mg separation. The solution’s structure is mainly reflected in its dynamic structure used to obtain information such as water molecule vibration and rotational relaxation time and its statistical structure for information such as water bond length, bond angle, solute hydration number and water molecule spatial distribution. The ionic solvation effect is one of the focuses of this paper, that is, the interaction between ions and water molecules. The research on ionic solvation is controversial. Marcus et al. [15] concluded that the influence of ions on water structure is remote; ions can enhance or destroy the hydrogen bond network, that is, the formation or destruction of the structure. Dill et al. [16] used Monte Carlo simulations to show that ionic effects on water hydrogen bonding are related to ionic charge density. Tielrooij et al. [17] used terahertz dielectric relaxation spectroscopy and polarization-resolved femtosecond infrared pumping spectroscopy to show that ionic effects on the hydration layer are related to the anionic and cationic hydration capacity and that the effects are predominantly on the first hydration layer. Geissler et al. [18], by a detailed comparison of experimental Raman spectroscopy measurements with classical MC simulations, determined that the ionic influence on hydrogen bonding is limited to the first solvation shell, with a weak influence outside the first hydration layer. Mitra et al. [19] used terahertz time-domain spectroscopy (TTDS) in the terahertz frequency region to study the effect of monovalent cations on the collective hydration dynamics of water, affirming that these ions are water structure disruptors. Havenith et al. [20] reported a series of divalent salts on terahertz absorption spectroscopy and molecular dynamics simulation studies of aqueous solutions, inferring that there is a synergistic effect of ions of different valences on the structural impact of water. H.J. Bakker et al. [21,22] pointed out that ions had little effect on the rotational dynamics of water molecules outside the first hydration shell, and ions did not destroy the hydrogen bond network structure of water.

For the study of ionic solvation, spectroscopy is a powerful means to obtain information on the solution’s microstructure. Fourier transform infrared (FTIR) spectroscopy and Raman spectroscopy are usually used to study the structure of hydration shells [23,24,25]. The O-H stretching vibration is very sensitive to the interaction between the solute and solvent molecules in an aqueous solution. Although the spectra of hydrated water and bulk water are different, the overlap is severe. To extract the spectral information of the hydration shell from the mixed spectrum of a solution, a certain data processing method must be adopted. Multivariable curve resolution (MCR) [23,26], factor analysis (FA) [27], ratio spectra [28] and fitting analysis [29] can also be used to directly extract the hydration spectra of solutes to obtain more direct information about the hydration structure. Excess spectroscopy applies the theory of excess functions in thermodynamics to the study of spectroscopy. It can enhance the spectroscopy of solvated water molecules and weaken the spectroscopy of bulk water molecules, thus providing more information about the interactions between molecules [30].

Liu et al. [31] studied the effects of different ions on the structure of methanol by using the excess Raman spectra of the O-H stretching peak. The results showed that in methanol electrolyte solutions with the same anions and different cations, the positive peaks of the excess Raman spectrum overlapped entirely, and the positive peaks of the excess Raman spectrum were solvent molecules affected by anions. Omta et al. [21] investigated the effect of ions on the hydrogen bond structure in liquid water by femtosecond pump detection spectroscopy. The results showed that the ions did not affect the rotational dynamics of the water molecules outside the first hydration shell. Näslund et al. [32] used X-ray absorption spectroscopy (XAS) and X-ray Raman scattering (XR) to study the influence of ionic solvation on the hydrogen bond structure of bulk water. The results showed that the effect of anions and cations was mainly on the first hydration shell. However, the ultrafast Kerr effect was used to study the low-frequency OH-X-hydrogen bond mode, indicating that Li^+^ is a monovalent ion, but the effect on the band frequency of the hydrogen bond model is even greater than that of Ca^2+^ [33]. Many previous studies have shown that Li^+^ has different properties than other alkali metal ions in terms of its hydration layer structure and hydration number [34,35].

In this paper, the Raman spectroscopy of a Li-Mg sulfate separation system at room temperature was systematically studied, which provided rich structural information on solute hydrate shells. Based on the changes in the positive and negative peak areas in the excess spectra of SO_4_^2−^ and O-H stretched vibration, the interaction of SO_4_^2−^ with water molecules and the structure of the hydrogen bond network in solution were discussed, respectively. The hydrated shell spectra in different solutions were extracted by Raman ratio spectra. The hydration structure of different solutions and the number of water molecules in the ionic hydration shell were studied via Raman spectroscopy of the hydration shell.

## 2. Results and Discussion

### 2.1. Raman Spectroscopy of Li_2_SO_4_-H_2_O System

#### 2.1.1. Raman Spectra and Excess Raman Spectra of SO_4_^2−^ Symmetric Stretching Vibration

**Figure 1:**
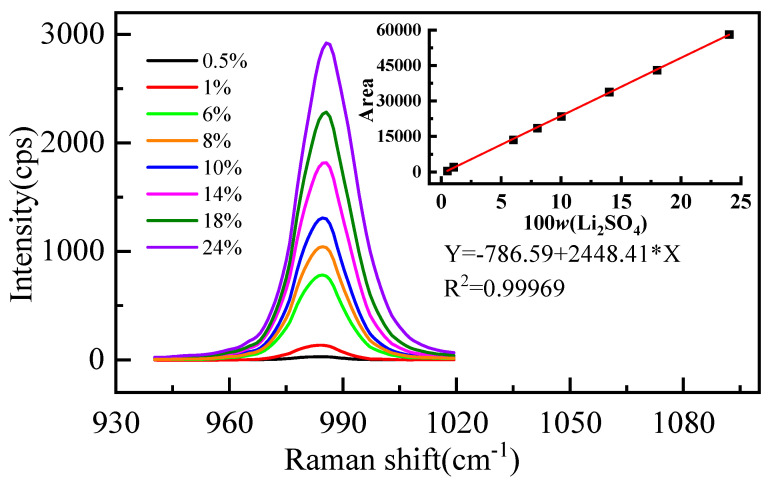
Characteristic peak spectra of SO_4_^2−^ in aqueous Li_2_SO_4_ solution with different mass fractions.

The measured Raman spectral signatures of Li_2_SO_4_ aqueous solutions for various mass fractions of Li_2_SO_4_ are shown in Figure 1. We found that the peak at 980 cm^−1^ increases with the increment of the mass fraction of Li_2_SO_4_ in the solution. Furthermore, a quantitative description of the relationship between the mass fraction and the integral area of the spectrum is shown in Figure 1 (*R*^2^ is the correlation coefficient). The integral area of the vibration peak Y is proportional to the mass fraction of Li_2_SO_4_ (w%), and the linear relationship is Y = −786.59 + 2448.41 × X. Significantly, with increasing concentration, the position of the spectral peak redshifts and the width increases. The position and width of the spectral peak should not change if the form of the ions in the solution does not change [36]. The observed changes should be attributed to various ion pairs at higher concentrations [36]. In a high-concentration solution, Li^+^ interacts with the oxygen atom of SO_4_^2−^, and the moving direction of the spectral peak is determined by the competition between the two forces. One is the strong interaction between Li^+^ and SO_4_^2−^, which weakens the strength of the S-O bond and causes a redshift of the spectral peak. The other is that Li^+^ destroys the hydrogen bond between SO_4_^2−^ and water molecules, which makes the SO_4_^2−^ fully symmetric stretching vibration peak blueshift.

**Figure 2:**
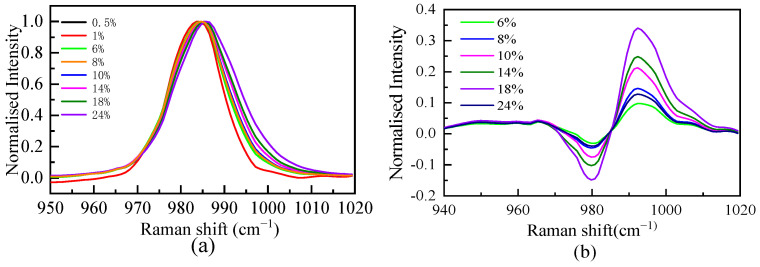
Normalized characteristic peak spectra (**a**) and excess Raman spectra (**b**) of SO_4_^2−^ in solutions with different mass fractions.

The spectral data of the SO_4_^2−^ fully symmetric stretching vibration interval are normalized, and the results are shown in Figure 2. As shown in Figure 2, the position of the spectral peak is redshifted with increasing mass fraction, indicating that the interaction between Li^+^ and SO_4_^2−^ in the solution makes the S-O bond longer. The Raman spectra of free hydrate ions need to be obtained when calculating the excess Raman spectra. Since the position and width of the peak are affected by the form of ion hydration, the intensity of the peak is determined by the ion concentration. The ratio of peak strength to maximum strength is used to normalize the SO_4_^2−^ symmetric stretching vibration. When the mass fractions were 0.5% and 1%, the Raman spectra of the SO_4_^2−^ symmetric stretching vibration (Figure 2) completely overlapped after normalization, indicating that at these two concentrations, the SO_4_^2−^ in the solution was mainly free ions. The 1% mass fraction of the SO_4_^2−^ symmetric stretching vibration Raman spectrum is considered the free hydrated ion spectrum (ideal spectrum). Based on the definition of the excess spectrum, the excess Raman spectrum was obtained by subtracting the spectrum of the 1% mass fraction solution from the experimental spectrum of the other concentrations, as shown in Figure 2. The excess Raman spectrum mainly presents one or two positive peaks and one negative peak. The negative peak represents the reduced free hydrated SO_4_^2−^ ions, and the positive peak represents the increased composition of the system compared with the ideal solution, namely, the ion pair. The peak position of the negative peak of the excess Raman spectrum at each concentration does not change with the concentration, and the peak position of the positive peak changes with the concentration, indicating that there is more than one form of ion pair.

#### 2.1.2. Raman and Excess Raman Spectra of the O-H Stretching Vibration Range

**Figure 3:**
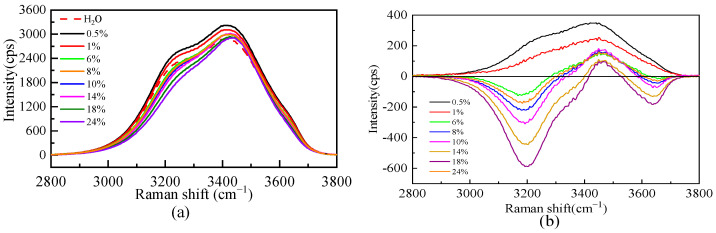
Raman spectra of O-H stretching vibration (**a**) and excess Raman spectra (**b**) of aqueous Li_2_SO_4_ solution at different mass fractions.

Due to the dissolution of salt, the microstructure of the aqueous solution will be affected. Figure 3 shows the Raman spectra of the O-H stretching vibration range of pure water and the Li_2_SO_4_ aqueous solution at different mass fractions. Figure 3 shows that the whole O-H stretching vibration region in a dilute solution (0.5% and 1%) is stronger than that in pure water. The whole spectrum is positive in the excess Raman spectrum, indicating that Li_2_SO_4_ in the dilute solution is conducive to promoting the construction of a water–hydrogen bond network structure, which is manifested as the “structure-making” solute. With an increasing Li_2_SO_4_ concentration, the intensity decreases at the shoulder peak of the Raman shift of 3200 cm^−1^ and increases at the main peak of 3400 cm^−1^. To further analyze the specific changes in the structure in the solution, the spectra were peaked to obtain the excess Raman spectrum. There is no free-ion spectrum in the vibration range, so the experimental spectrum deducted from the pure water Raman spectrum is the excess Raman spectrum, as shown in Figure 3. There are positive and negative peaks in the excess Raman spectra, in which the negative peak represents the reduced bulk water. With increasing salt concentration, more volumetric water structures are destroyed, which is manifested by the increase in the integral area of the negative peak in the excess spectra with increasing salt concentration. The positive peak in the excess Raman spectrum represents the newly increased component in the relative ideal system, namely, the water molecule interacting with ions, namely, the increase of the ionic hydrated water molecule, and its intensity decreases with the increase in concentration. The amount of water of hydrated by SO_4_^2−^ decreases as the concentration increases.

**Figure 4:**
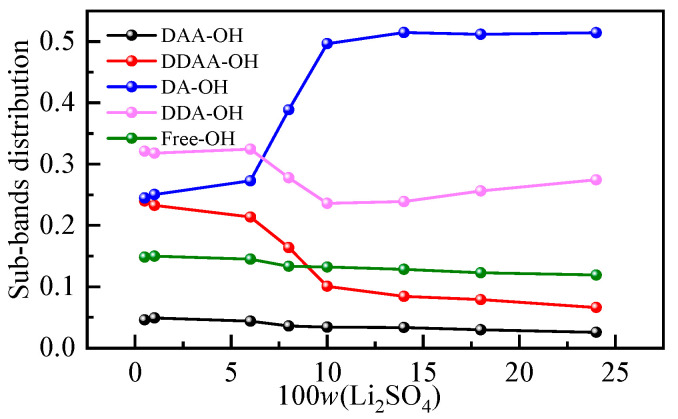
Hydrogen bond structures in aqueous Li_2_SO_4_ solutions with different mass fractions.

It is generally recognized that the Raman spectra of the O-H stretching band of water at 293 K and 0.1 MPa can be decomposed into five subpeaks, which are concentrated at approximately 3062, 3207, 3328, 3447 and 3580 cm^−1^ [37,38]. According to the proton donor (D is the donor, A is the receptor), these peaks are defined as the DAA (single donor-double acceptor), the DDAA (double donor-double acceptor), the DA (single donor-single acceptor), the DDA (double donor-single acceptor) and free OH stretching vibrations. The low-frequency O-H stretching subband is mainly attributed to water molecules with strong hydrogen bonds, while the high-frequency subband corresponds to water molecules with weak hydrogen bonds [39]. To further analyze the specific changes in the hydrogen bond structure in a solution, the O-H vibration peak was divided into smaller peaks. Figure S1 shows the peak diagram of the Li_2_SO_4_ aqueous solution with different mass fractions in the O-H stretching vibration range, and the change in the relative integral area shows the change in the proportion of five structures in the aqueous solution structure. The integral area and corresponding occupancy data for different subbands are listed in Table S1. The proportion of five hydrogen-bonding structures in an aqueous solution varies with mass fraction, as shown in Figure 4. It can be seen from Figure 4 that with the increase in the mass fraction of the Li_2_SO_4_ solution, the proportion of DAA and DDAA hydrogen-bonding structures at low wavenumbers in the overall structure gradually decreases, while the proportion of DA hydrogen-bonding structures at 3300 cm^−1^ increases when the increase in mass fraction is greater than 6.00%, and the overall trend changes little after the sudden increase.

#### 2.1.3. Hydrate Shell Spectrum and Hydration Number

The spectrum of the hydration shell directly reflects the structural and spectral properties of water molecules affected by the solute. Through an analysis of the hydration layer spectra, the structural information of the interaction between solute and water molecules, the structure of the hydration shell and the number of water molecules can be obtained [9]. The spectra of the solutions with different concentrations can be divided into solute spectra, hydrated shell water molecular spectra and bulk water spectra.

(1) $$I_{\mathrm{solution}}(\nu )=I_{\mathrm{bulk}}(\nu )+I_{\mathrm{hydration}}(\nu )+I_{\mathrm{solute}}(\nu )$$

In Equation (1), *I* and *ν* are the spectral intensity and frequency, respectively. Due to the different numbers of water molecules involved in Raman scattering under the same light-irradiation volume, the spectral intensities of the bulk water spectrum and pure water spectrum are different, but the spectral shapes are consistent. Equation (1) can be rewritten as follows:

(2) $$I_{\mathrm{solution}}(\nu )=AI_{\mathrm{pure}}(\nu )+I_{\mathrm{hydration}}(\nu )+I_{\mathrm{solute}}(\nu )$$

The stretching vibration peak of O-H in the 2800–3800 cm^−1^ spectrum can sensitively express the microstructure of an aqueous solution. Many research results show that due to the localization of the influence of ions on water, the water molecules on the hydration shell at a low frequency (<3100 cm^−1^) have no vibration peak [12], and there are bulk water and hydration water molecules affected by ions in the solution. Ions limit the vibration mode of hydrogen bonds in hydrated water, and the O-H stretching vibration spectrum is narrower than that of pure water. In the common inorganic salt solution system, there is no vibration peak of solute ions in the O-H stretching vibration region.

(3) $$A=I_{\mathrm{solution}}(\nu <3100)/I_{\mathrm{pure}}(\nu <3100)$$

(4) $$I_{\mathrm{hydration}}(\nu )=I_{\mathrm{solution}}(\nu )-AI_{\mathrm{pure}}(\nu )$$

**Figure 5:**
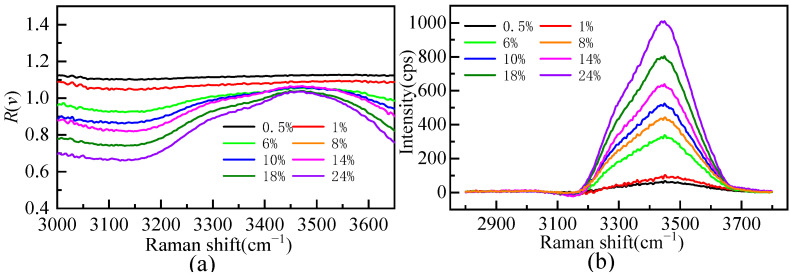
The Raman ratio spectra (**a**) and Raman spectrum of hydration shell in OH stretching vibration region (**b**) of aqueous Li_2_SO_4_ solution at different mass fractions.

Figure 5a shows the Raman ratio spectra of different mass fractions of the Li_2_SO_4_ solution compared to that of pure water. There is a plateau in the low-wavenumber segment of the Raman ratio spectra; that is, the Raman ratio spectra intensity is constant. The solution spectrum is parallel to the pure water spectrum, and the hydrate water molecule does not contribute to this range. The concentration of the Li_2_SO_4_ solution changed from dilute to concentrated, and the factor *A* value changed from 1.10 to 0.67, indicating that there is still a large proportion of bulk water molecules in the Li_2_SO_4_ solution at near-saturated concentrations. When the mass fractions of the Li_2_SO_4_ solution were 0.005 and 0.01, the factor *A* value was greater than one, indicating that Li_2_SO_4_ could enhance the Raman spectrum intensity of bulk water molecules at a very low concentration. This proved that Li_2_SO_4_ in dilute solution was conducive to promoting water hydrogen bond network structure construction, manifested as a “structure-making” solute. In this work, the hydrated shell spectra of Li_2_SO_4_ from extremely dilute to concentrated aqueous solutions were extracted by Raman ratio spectra, and the hydrated shell spectra of SO_4_^2−^ at various mass fractions were obtained, as shown in Figure 5b. Figure 5b shows that the hydration shell spectrum is zero before 3100 cm^−1^, and the hydration shell spectrum mainly comes from the Raman spectrum of water molecules in the SO_4_^2−^ hydration shell [21,31,32,33].

The hydration shell spectrum can also provide more information about the hydration number of ions and be used to calculate the number of water molecules in the hydration shell. The hydration number of a single solute (ion and molecule) is given as follows:

(5) $$N_{\mathrm{hydration}}=\frac{n_{\mathrm{hydration}}}{n_{\mathrm{solute}}}=\frac{n_{\mathrm{hydration}}}{n_{\mathrm{water}}}/\frac{n_{\mathrm{solute}}}{n_{\mathrm{water}}}$$

(6) $$n_{\mathrm{water}}=n_{\mathrm{hydration}}+n_{\mathrm{bulk}}=\frac{\int I_{\mathrm{hydration}}(\nu )d\nu }{k\sigma_{\mathrm{hydration}}}+\frac{\int I_{\mathrm{bulk}}(\nu )d\nu }{k\sigma_{\mathrm{water}}}$$

*I*(*ν*) is the spectrum of water molecules on the hydration shell and the spectrum of bulk water, respectively. *σ* is the Raman scattering cross section, and *k* is the coefficient. Substituting Formula (6) into Formula (5), we obtain

(7) $$N_{\mathrm{hydration}}=\frac{\int I_{\mathrm{hydration}}(\nu )d\nu }{\int I_{\mathrm{hydration}}(\nu )d\nu +\frac{\sigma_{\mathrm{hydration}}}{\sigma_{\mathrm{water}}}\int I_{\mathrm{bulk}}(\nu )d\nu }/\frac{n_{\mathrm{solute}}}{n_{\mathrm{water}}}$$

The $\sigma_{\mathrm{hydration}}$ and $\sigma_{\mathrm{water}}$ values are different for different salt solutes, and the $\sigma_{\mathrm{hydration}}/\sigma_{\mathrm{water}}$ values can be accurately calculated for each concentration according to the Raman scattering cross-section ratio and then taken as the average. The cross-section ratio of the Raman scattering at different concentrations is basically unchanged, and only small changes can be considered errors. $\sigma_{\mathrm{hydration}}/\sigma_{\mathrm{water}}$(SO_4_^2−^) = 0.99 was given in reference [28].

**Figure 6:**
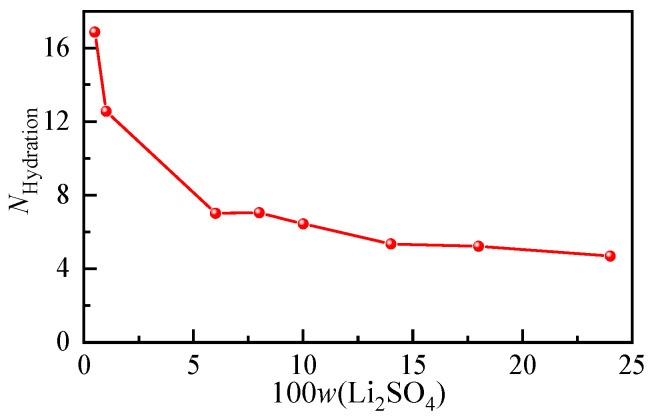
Concentration-dependent ion hydration number of aqueous Li_2_SO_4_ solutions with different mass fractions.

According to the hydration shell spectra at different concentrations of the Li_2_SO_4_ solution, the anionic hydration number at each concentration was calculated, and their variation trends with increasing solution concentration are shown in Figure 6. In the Li_2_SO_4_ solution with a mass fraction of 0.01, the hydration number of SO_4_^2−^ was determined to be 12.56. According to the results of the Raman spectroscopy [28] and MD simulation [40], the first hydration of SO_4_^2−^ was also consistent with 12.0 ± 0.2 and 11, respectively. From Figure 6, it can be seen that the number of water molecules in the SO_4_^2−^ hydration shell decreases significantly with increasing concentration, and the hydration number decreases from 16.86 to 4.7 when the dilute mass fraction is 0.005 to nearly saturated at 0.24. Even in the dilute concentration range, the hydration number decreased from 16.86 to 12.56 from *w* = 0.005 to *w* = 0.01. The transformation of solvent-shared ion pairs (SSIPs) from free hydrated anions to contact ion pairs (CIPs) or even clusters occurs.

### 2.2. Raman Spectroscopic for Li_2_SO_4_-MgSO_4_-H_2_O System

#### 2.2.1. Raman Spectra of O-H Stretching Vibration Range

**Figure 7:**
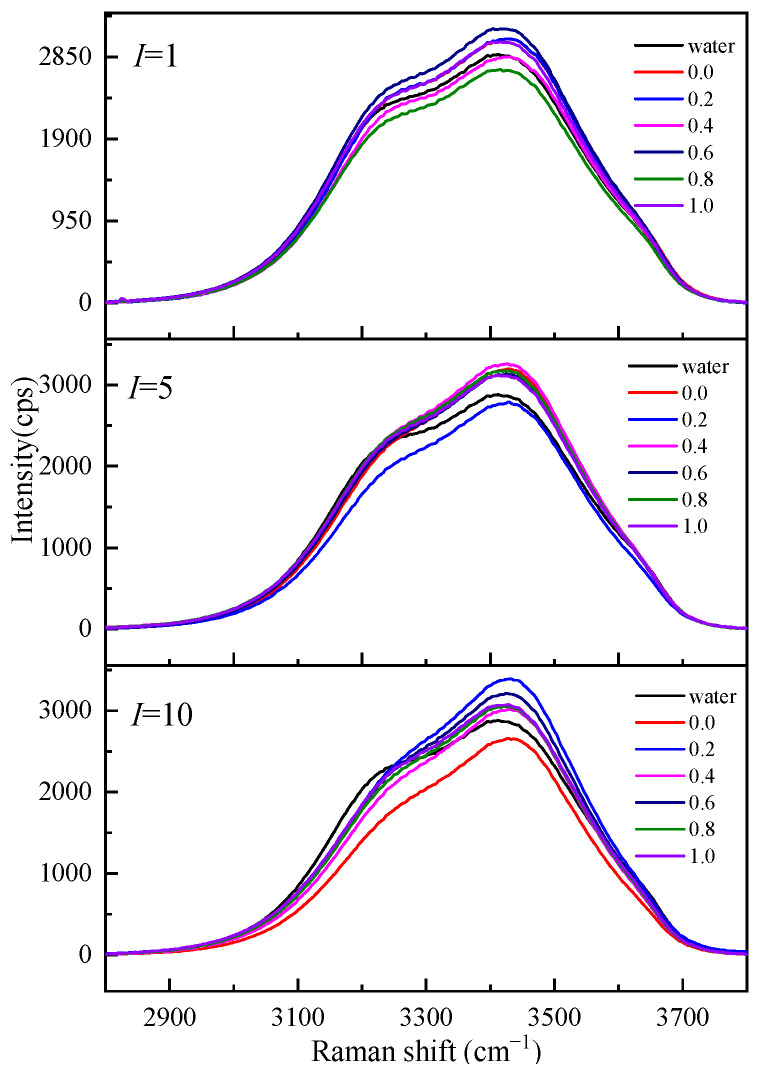
Raman spectra of aqueous Li_2_SO_4_-MgSO_4_ solutions at various ionic strength ratios.

To understand the influence of different cations on the O-H stretching vibration Raman spectra in the Mg-Li separation system, we studied the O-H stretching vibration Raman spectra of Li_2_SO_4_-MgSO_4_ mixed solutions with different ionic strengths and strength ratios, as shown in Figure 7. Figure 7 shows that the shoulder peaks at low wavenumbers (3200 cm^−1^) gradually disappear with increasing ionic strength, while the strength at high wavenumbers increases with increasing ionic strength. The Raman vibration at low wavenumbers is mainly the O-H vibration of bulk water, while the O-H vibration peak affected by ions in the solution is mainly at high wavenumbers. In the same ionic strength solution, the Raman spectrum intensity of the solution with high Li^+^ ionic strength at low wavenumbers is much lower than that of the solution with high Mg^2+^ ionic strength. The effect of Li^+^ ions with the same ionic strength on water is more significant than that of Mg^2+^ ions, and the number of water molecules in a solution with a high Li^+^ ionic strength is relatively small.

#### 2.2.2. Hydration Shell Spectrum and Hydration Number

**Figure 8:**
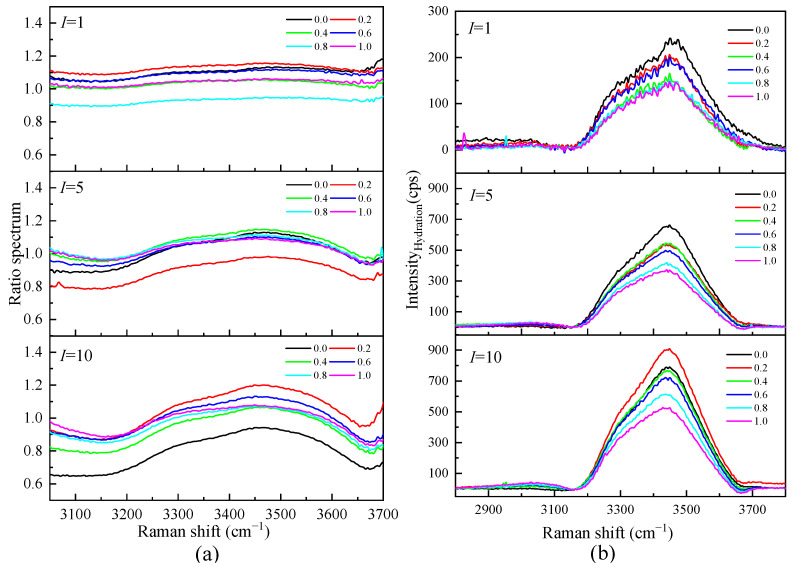
(**a**)The Raman Ratio spectra of mixed aqueous Li_2_SO_4_-MgSO_4_ solution and the corresponding spectra of the hydration shell (**b**) in OH stretching vibration region.

Figure 8a shows the Raman ratio spectra of each ionic strength in the mixed solution of Li_2_SO_4_ and MgSO_4_. Under different ionic strengths, there is a plateau in the low-wavenumber section; that is, the ratio spectra intensity is constant. In this frequency range, the solution spectrum is parallel to the pure water spectrum, and the hydration shell water molecules do not contribute to this range. The ratio spectra factor *A* ranged from 1.0825 to 0.6579 in the mixed solutions with different ionic strengths, indicating that there was still a large proportion of bulk water molecules in the high-concentration solution with ionic strength *I* = 10 mol·kg^−1^. By distinguishing the hydration shell spectrum from the body water spectrum, it can be proven that the influence of ions on the water–hydrogen bond network is localized. The influence of solute ions on the water molecules in the first hydration shell is significant, while the influence on the water molecules in the outer layer of the hydration shell is weak. It can also be seen from Figure 8b that in the mixed solution with the same total ionic strength, the hydration spectrum decreases with the increase in the proportion of MgSO_4_, indicating that Li_2_SO_4_ has a more significant impact on water molecules than MgSO_4_ in the same ionic strength solution. This is mainly because Li^+^ has a strong hydration effect. In addition to the first hydration shell water molecules, the second coordination shell water molecules cannot be ignored.

**Figure 9:**
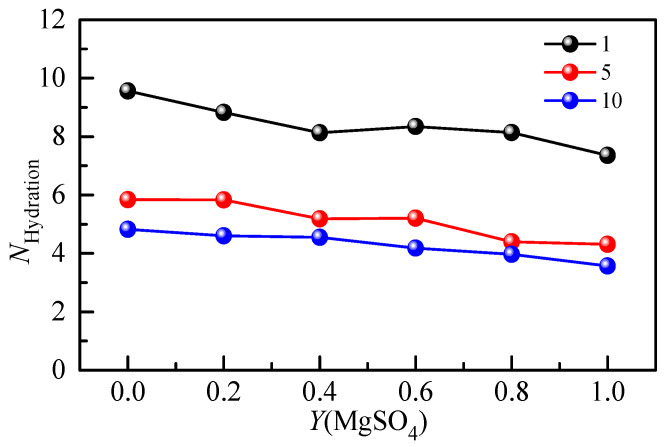
Concentration-dependent hydration number of mixed aqueous Li_2_SO_4_-MgSO_4_ solution with different ionic strengths.

The hydration number of SO_4_^2−^ in the Li_2_SO_4_-MgSO_4_ mixed solution was calculated by Raman spectroscopy of the hydration shell. Figure 9 shows the hydration number of SO_4_^2−^ with the MgSO_4_ ionic strength ratio under different ionic strengths. The hydration number of SO_4_^2−^ in the 1 mol·kg^−1^ ionic strength solution was less than that in the dilute solution (*w* = 0.005), indicating that ionic pairs appeared in the 1 mol·kg^−1^ ionic strength solution. In a highly concentrated mixed-salt solution, most of the water molecules form hydrated shells around SO_4_^2−^, and the reduction in the number of bulk water molecules is not conducive to the formation of large, free, hydrated ion hydrate clusters. The hydration number decreased gradually with increasing salt ionic strength, indicating that the degree of ion aggregation increased. The free hydration ions in the solution would form shared ion pairs (SIPs) and contact ion pairs (CIPs) with cations. In the mixed solution, the anion hydration number decreases gradually with the increase in the MgSO_4_ ion strength fraction, indicating that different cations significantly affect the anion hydration structure. Since Mg^2+^ ions are divalent ions with a stronger hydration capacity than monovalent Li^+^ ions, there is a strong interaction between Mg^2+^ and SO_4_^2−^ ions, which leads to the aggregation of ions and the formation of ion pairs.

## 3. Experimental

### 3.1. Sample Preparation

The chemical reagents used in this work are listed in Table 1, including the sources, final purity and impurity analysis methods. The stock solution was prepared by dissolving recrystallized Li_2_SO_4_·H_2_O and MgSO_4_·7H_2_O (McLean Biochemical Technology Co., Ltd., Shanghai, China) with a purity not less than 99.95%. In the experiment, all deionized water was obtained from an ultrapure water mechanism (Sichuan Youpu Ultrapure Technology Co., Ltd., Chengdu, China), and the resistivity of the deionized water was 18.25 MΩ cm at room temperature. Near-saturated Li_2_SO_4_ and MgSO_4_ stock solutions were prepared, and the molality of stock solutions was determined using the barium-sulfate-burning gravimetric method. The physicochemical properties of Li_2_SO_4_ solutions and ternary mixed solutions are listed in Table 2 and Table 3, respectively.

### 3.2. Experimental Method

The Raman spectra were measured by a confocal Raman microscope (Xplo RA Microscope, Horiba Jobin Yvon, Paris, France) under a backscattering configuration with an excitation laser wavelength of 532 nm and a 50× objective lens. The diffraction grating with 400 lines/mm provided a spectral resolution of less than 1 cm^−1^. Frequency calibration of the Raman spectrum was realized using the characteristic 520 cm^−1^ lines of silicon. The incident laser power on the samples was approximately 10 mW. All spectra were collected using a 15 s measurement time and 20 accumulations.

## 4. Conclusions

Raman spectroscopy was used to study the changes in the hydrogen bond structure and the interaction between ions in an aqueous Li_2_SO_4_ solution and Li_2_SO_4_-MgSO_4_ mixed solution. The SO_4_^2−^ symmetric stretching vibration peak at 940~1020 cm^−1^ and the O-H stretching vibration peak at 2800~3800 cm^−1^ of the aqueous Li_2_SO_4_ solution at room temperature were studied via Raman spectroscopy and excess spectroscopy. The integral area of the positive and negative peaks of the excess Raman spectroscopy was analyzed, and the change in the hydrogen bond network structure in the aqueous solution was studied. When the mass fraction was less than 1%, Li_2_SO_4_ showed ‘structure-making’ and has an inconspicuous secondary hydration shell. When the mass fraction was greater than 1%, ion pairs gradually began to appear in the solution. In the mixed solution, the anion hydration number decreased gradually with the increase in the MgSO_4_ ion strength fraction, indicating that different cations significantly affect the anion hydration structure. Since Mg^2+^ ions are divalent ions with a stronger hydration capacity than monovalent Li^+^ ions, there is a strong interaction between Mg^2+^ and SO_4_^2−^ ions, which leads to the aggregation of ions and the formation of ion pairs.

The Raman ratio spectra were employed to extract the hydration shell spectra of the solute from the solution spectrum. The hydration shell spectra of SO4^2−^ from the diluted solution and the concentrated solution were employed to study the structures of the hydration shell. The interaction between the solute and water molecules, the structure of the hydration shell and the number of water molecules were obtained by analyzing the spectra of these hydration shells. The spectra of the hydration shell was employed to quantitatively calculate the number of water molecules in the first hydration shell. It was found that the number of water molecules in the hydration shell decreased with an increasing concentration, and ion pairs began to form in the Li_2_SO_4_ solution after the mass fraction was greater than 1%. In the mixed solution of Li_2_SO_4_ and MgSO_4_, different cations and anions have different hydration numbers, indicating that there is a strong interaction between ions in the mixed solution, resulting in ion aggregation and the formation of ion pairs. As the concentration of the salt solution increases, the hydration number decreases rapidly, indicating that the degree of ion aggregation increases with increasing concentration. The results show that the Raman ratio spectra method can be used to study the microstructure and kinetics of aqueous solutions of lithium–magnesium separation systems.

## Figures and Tables

**Table 1:** Chemical reagent description table.

Compound	Source	Final Purity	Impurity Analysis Method
Li_2_SO_4_·H_2_O	McLean Biochemical Technology Co., Ltd.	0.995	ICP
MgSO_4_·7H_2_O	McLean Biochemical Technology Co., Ltd.	0.995	ICP
BaCl_2_	Sinopharm Chemical Reagent Co., Ltd. (Shanghai, China)	A.R.	--
Water	Double distillation	1.5 × 10^−4^ S·m^−1^	Conductivity meter

**Table 2:** Mass fraction and molar ratio of Li_2_SO_4_ aqueous solution ^a^.

No. ^a^	100*w*(Li_2_SO_4_)	*ρ*/(g·cm^−3^)	*m*(mol·kg^−1^)	*n*(Li_2_SO_4_):*n*(H_2_O)
1	0.5	1.00257	0.04584	0.0008258
2	1.0	1.00702	0.09343	0.0016831
3	6.0	1.05047	0.5814	0.0104739
4	8.0	1.06820	0.7962	0.0142517
5	10.0	1.08593	1.0110	0.0182143
6	14.0	1.12227	1.4806	0.0266741
7	18.0	1.15968	1.9962	0.0359628
8	24.0	1.21748	2.8738	0.0517729

^a^ Standard uncertainties, *u*, are *u*(*ρ*) = 0.1 mg·cm^−3^ and *u*(*m*) = 0.00004 mol·kg^−1^.

**Table 3:** Ionic strength and molar ratio of Li_2_SO_4_-MgSO_4_-H_2_O aqueous solution.

No. ^a^	*I*(mol·kg^−1^)	*Y*(MgSO_4_)	*m*Li_2_SO_4_(mol·kg^−1^)	*m*MgSO_4_(mol·kg^−1^)	*n*(solute):*n*(H_2_O)
1	1	0.0	0.3335	0.0000	0.00600796
2	1	0.2	0.2666	0.0548	0.00579009
3	1	0.4	0.2012	0.0984	0.00539825
4	1	0.6	0.1328	0.1504	0.00510299
5	1	0.8	0.0672	0.2006	0.00482338
6	1	1.0	0.0000	0.2489	0.00448382
7	5	0.0	1.3318	0.0000	0.02399285
8	5	0.2	1.0656	0.2027	0.02284913
9	5	0.4	0.8005	0.3990	0.02160967
10	5	0.6	0.5330	0.6016	0.02044111
11	5	0.8	0.2665	0.8003	0.01921944
12	5	1.0	0.0000	1.0015	0.01804287
13	10	0.0	2.3342	0.0000	0.04205187
14	10	0.2	1.8659	0.3500	0.03992034
15	10	0.4	1.3986	0.6979	0.03776931
16	10	0.6	0.9333	1.0488	0.03570847
17	10	0.8	0.4687	1.3977	0.03362393
18	10	1.0	0.0000	1.7526	0.03157351

^a^ Standard uncertainties *u*(*m*) = 0.00004 mol·kg^−1^.

## Data Availability

Not applicable

## Supplementary Materials

The following supporting information can be downloaded at https://www.mdpi.com/article/10.3390/molecules28217356/s1: Figure S1: Peak splitting data of aqueous Li_2_SO_4_ solution with different mass fractions in O-H stretching vibration interval. Table S1: Sub-bands integral area and occupy of aqueous Li_2_SO_4_ solution with different mass fractions in O-H stretching vibration interval.
