# Supplementary material for: Raman Spectrum of the Li2SO4-MgSO4-H2O System: Excess Spectrum and Hydration Shell Spectrum
Source: Molecules. 2023 Oct 31;28(21):7356. doi: 10.3390/molecules28217356 (PMC10648143; doi:10.3390/molecules28217356)
Supplement: Supplementary file 1 [file molecules-28-07356-s001.zip › molecules-2663127-supplementary.pdf]

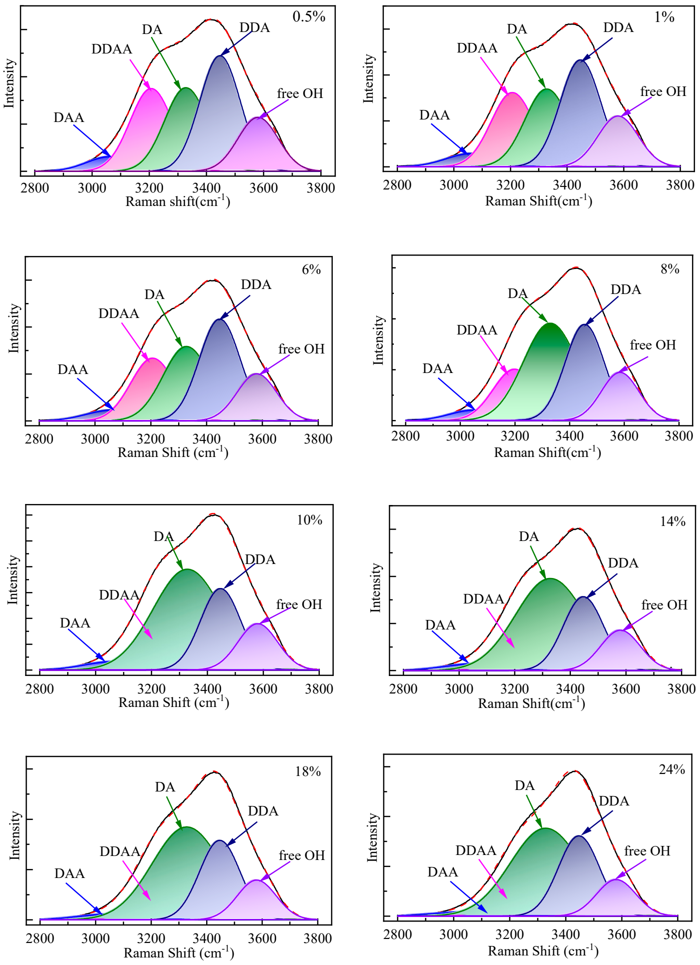


**Figure S1.** Peak splitting data of aqueous $Li_2SO_4$ solution with different mass fractions in O-H stretching vibration interval

**Table S1.** Sub-bands integral area and occupy of aqueous $Li_2SO_4$ solution with different mass fractions in O-H stretching vibration interval.

| *c*(%) | total area | sub-bands1 | | sub-bands2 | | sub-bands3 | | sub-bands4 | | sub-bands5 | |
|---|---|---|---|---|---|---|---|---|---|---|---|
| | | area | occupy | area | occupy | area | occupy | area | occupy | area | occupy |
| 0.05 | 1356083.5 | 62571.9 | 0.0461 | 325330.8 | 0.2399 | 331872.1 | 0.2447 | 435236.0 | 0.3210 | 201072.7 | 0.1483 |
| 1.0 | 1314630.3 | 64543.0 | 0.0491 | 306006.7 | 0.2328 | 329020.8 | 0.2503 | 417981.4 | 0.3179 | 197078.4 | 0.1499 |
| 6.0 | 1230676.7 | 53968.8 | 0.0439 | 263040.3 | 0.2137 | 335824.5 | 0.2729 | 399490.0 | 0.3246 | 178353.1 | 0.1449 |
| 8.0 | 1217929.9 | 44044.0 | 0.0362 | 199650.8 | 0.1639 | 473175.2 | 0.3885 | 338370.7 | 0.2778 | 162689.2 | 0.1336 |
| 10.0 | 1205320.8 | 41344.6 | 0.0343 | 121585.6 | 0.1009 | 598287.0 | 0.4964 | 284763.3 | 0.2363 | 159340.3 | 0.1322 |
| 14.0 | 1189145.7 | 39911.0 | 0.0336 | 100192.0 | 0.0843 | 611870.0 | 0.5145 | 284221.7 | 0.2390 | 152951.0 | 0.1286 |
| 18.0 | 1127717.7 | 33817.4 | 0.0300 | 89083.5 | 0.0790 | 577047.4 | 0.5117 | 289027.6 | 0.2563 | 138741.8 | 0.1230 |
| 24.0 | 1081347.8 | 27825.6 | 0.0257 | 71810.0 | 0.0664 | 556026.6 | 0.5142 | 296743.3 | 0.2744 | 128942.4 | 0.1192 |
